# Reversing the Natural Drug Resistance of Gram-Negative Bacteria to Fusidic Acid via Forming Drug–Phospholipid Complex

**DOI:** 10.3390/bioengineering11020177

**Published:** 2024-02-11

**Authors:** Jianhong Liu, Xuyang Lai, Yuanhong Li, Zhuohang Yu, Xuan Wang, Chaoliang Zhang, Qiang Peng

**Affiliations:** State Key Laboratory of Oral Diseases & National Center for Stomatology & National Clinical Research Center for Oral Diseases, West China Hospital of Stomatology, Sichuan University, Chengdu 610041, China

**Keywords:** drug delivery, drug resistant, phospholipid complex, antimicrobial, antibiotics

## Abstract

Drug resistance substantially compromises antibiotic therapy and poses a serious threat to public health. Fusidic acid (FA) is commonly used to treat staphylococcal infections, such as pneumonia, osteomyelitis and skin infections. However, Gram-negative bacteria have natural resistance to FA, which is almost restrained in cell membranes due to the strong interactions between FA and phospholipids. Herein, we aim to utilize the strong FA–phospholipid interaction to pre-form a complex of FA with the exogenous phospholipid. The FA, in the form of an FA–phospholipid complex (FA-PC), no longer interacts with the endogenous membrane phospholipids and thus can be delivered into bacteria cells successfully. We found that the water solubility of FA (5 µg/mL) was improved to 133 µg/mL by forming the FA-PC (molar ratio 1:1). Furthermore, upon incubation for 6 h, the FA-PC (20 µg/mL) caused a 99.9% viability loss of *E. coli* and 99.1% loss of *P. aeruginosa*, while free FA did not work. The morphology of the elongated bacteria cells after treatment with the FA-PC was demonstrated by SEM. The successful intracellular delivery was shown by confocal laser scanning microscopy in the form of coumarin 6-PC (C6-PC), where C6 served as a fluorescent probe. Interestingly, the antibacterial effect of the FA-PC was significantly compromised by adding extra phospholipid in the medium, indicating that there may be a phospholipid-based transmembrane transport mechanism underlying the intracellular delivery of the FA-PC. This is the first report regarding FA-PC formation and its successful reversing of Gram-negative bacteria resistance to FA, and it provides a platform to reverse transmembrane delivery-related drug resistance. The ready availability of phospholipid and the simple preparation allow it to have great potential for clinical use.

## 1. Introduction

Bacterial infections, especially drug-resistant bacteria-induced ones, are a serious threat to human health and have caused many deaths [1,2,3]. Antibiotic therapy is the most important strategy to combat pathogenic bacteria. Unfortunately, the inappropriate use of antibiotics has led to the emergence of drug-resistant bacteria, which highly compromise the therapeutic efficacy [4,5,6,7]. In addition to the acquired drug resistance, some bacteria also have intrinsic resistance to a specific type of antibiotics.

Fusidic acid (FA), a steroidal antibiotic, is commonly used for the treatment of staphylococcal infections, including skin, bone and joint infections [8,9,10]. The antibacterial mechanism of FA is based on its irreversible binding to the elongation factor G (EF-G) located on the ribosome [11,12,13,14]. Consequently, the translocation of the nascent polypeptide chain from the A site to the P site, the formulation of the peptide bond and the release of the ribosome complex on reaching the stop codon are all blocked [15,16,17,18]. However, FA only has potent antibacterial activity against Gram-positive bacteria, and Gram-negative pathogens have natural resistance to FA [19,20,21]. It is reported that FA tends to be captured in the membrane of Gram-negative bacteria cells, due largely to its strong interactions with phospholipids in the membranes [22,23]. Another issue with FA is its poor water solubility. As we know, appropriate water solubility is of great importance for the convenient delivery of drugs. However, FA has a low water solubility due to its steroidal structure (5.2 μg/mL [24]). These issues highly limit the clinical applications of FA. As such, developing an approach for simultaneously enhancing FA solubility and reversing the natural resistance of Gram-negative pathogens is of great importance and significance. 

The drug–phospholipid complex (Drug-PC) is a physical composite formed by the interactions of drugs with phospholipids, basically by hydrogen bonding [25,26,27]. It has been well documented that the Drug-PC is highly effective in improving the solubility and bioavailability of the associated drugs [28,29,30,31,32,33]. For instance, the solubility of insulin in water was significantly enhanced from 59 µg/mL to 1150 µg/mL by forming an insulin–phospholipid complex (molar ratio 1:80) [31]. Due to these benefits, pre-formation of the Drug-PC between FA and an exogenous phospholipid may be an effective way to simultaneously enhance FA solubility and reverse the natural FA resistance of Gram-negative bacteria, and such an idea has never been reported. 

**Scheme 1:**
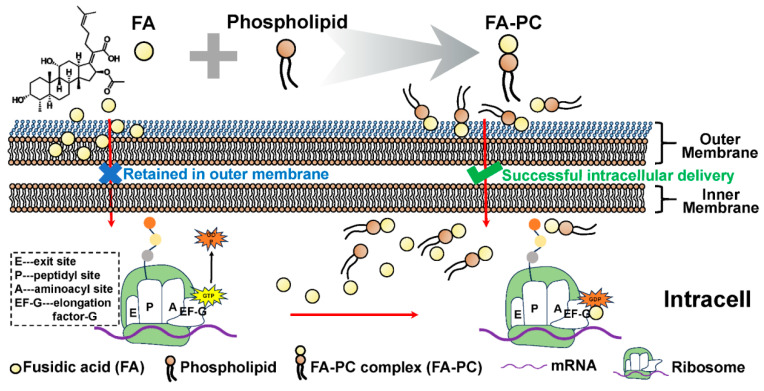
Schematic illustration of the formation of the fusidic acid–phospholipid complex (FA-PC) and its intracellular transportation into Gram-negative bacteria cells. Free FA is insoluble in water and commonly restrained in Gram-negative bacteria cell membranes due to the strong interactions between FA and membrane phospholipids. FA-PC is easily formed by physically compositing FA with exogenous phospholipid, which not only enhances the solubility of FA but also facilitates the intracellular delivery of FA.

In this work, inspired by the strong interaction of FA with the endogenous membrane phospholipid and the benefits of the phospholipid complex, we aim to pre-form the FA-phospholipid complex (FA-PC) by compositing FA with an exogenous phospholipid and systematically investigate the properties and antibacterial activity of the FA-PC against two representative Gram-negative bacteria (*E. coli* and *P. aeruginosa*). It is hypothesized that the FA-PC can significantly enhance the water solubility of FA due to the solubilization capacity of phospholipids. More importantly, FA, in the form of the FA-PC, no longer interacts with the endogenous phospholipids in the bacteria cell membrane, since the reaction sites of FA have already been occupied by the exogenous phospholipid in the FA-PC. As a result, the FA-PC is not captured in the bacterial cell membrane but can be successfully delivered into the bacteria cells, ultimately reversing the natural resistance of Gram-negative pathogens to FA (Scheme 1).

## 2. Materials and Methods

### 2.1. Materials

Phospholipid (soybean lecithin containing 90.4% phosphatidylcholine) was purchased from Shanghai Tai-wei Pharmaceutical Co. Ltd. (Shanghai, China). Fusidic acid was obtained from Shanghai Aladdin Biochemical Technology Co. Ltd. (Shanghai, China). Trifluoroacetic acid (TFA, analytical grade) and Coumarin 6 (C6) were purchased from Sigma-Aldrich (St. Louis, MO, USA). Luria–Bertani Medium was supplied by Coolaber Science & Technology Co., Ltd. (Beijing, China). DAPI staining solution (C1006) was purchased from Beyotime Biotech Inc. (Shanghai, China). 

### 2.2. Preparation of FA-PC 

The FA-PC was prepared according to previous methods with some minor modifications [28,29]. In detail, 13 mg of FA and 20 mg of phospholipid (molar ratio 1:1) were co-dissolved in 1 mL of absolute ethanol under magnetic stirring at room temperature. The mixture solution was further stirred at 55 °C for 2 h to yield a clear and transparent solution. The above solution was then subjected to rotary evaporation at 55 °C for 30 min under vacuum to remove the solvent. The dried FA-PC was sealed and stored at −20 °C immediately after evaporation until further use.

### 2.3. Characterization of FA-PC

#### 2.3.1. High-Performance Liquid Chromatography (HPLC)

The analysis was conducted using an HPLC system (Thermo Scientific, UltiMate 3000, Waltham, Mass, USA) equipped with a C18 (Acclaim^TM^ RSLC 120, Thermo Scientific, UltiMate 3000, Waltham, Mass, USA) analytical column (2.2 µm, 120 Å, 2.1 × 100 mm). The mobile phase consisted of acetonitrile and 0.1% TFA at a ratio of 80:20 (*v*/*v*). The system was run at a flow rate of 0.3 mL/min, with the column temperature at 30 °C. The detection was performed at the wavelength of 215 nm, and the injection volume was 1 µL. 

The samples were prepared as follows: deionized water was added to the prepared FA-PC, followed by constant shaking under 100 rpm and 25 °C for 24 h. Subsequently, the resultant liquid was centrifuged at 12 krcf and 4 °C for 5 min to separate the undissolved drug. Then, the supernatant was collected and diluted with the mobile phase, and a 10 µL aliquot of the solution was injected into the HPLC system. In addition, FA powder was also dissolved in 1 mL of mobile phase, and a 10 µL aliquot of the solution was injected into the HPLC system.

#### 2.3.2. The UV Spectroscopy

The samples (FA-PC, FA and PC) were dissolved in absolute ethanol and added to the 96-well plates, respectively. The UV absorption spectra within the wavelength range of 200–400 nm were recorded using the Multiskan Sky Microplate Spectrophotometer (Thermo Scientific, USA). The solvent absolute ethanol was used as the blank.

#### 2.3.3. The FT-IR Spectroscopy

The FTIR spectra of FA-PC, FA and PC were measured by Attenuated Total Reflectance FTIR spectroscopy (ATR-FTIR, Spectrum Two FT-IR Spectrometer, PerkinElmer, USA). In brief, the sample was dissolved in absolute ethanol, and 10 μL of the resultant solution was added on the testing board. After air-flow drying, the specimen was examined by ATR-FTIR in the range between 400 and 4000 cm^−1^.

### 2.4. Solubility

The water solubility of the FA-PC was examined by HPLC. In brief, the HPLC-based calibration was established by measuring the peak areas of FA standard samples (2.5, 5, 10, 20 and 40 µg/mL). The linear regression equation was obtained and used for the quantification of FA content. The prepared FA-PC (at molar ratios of 1:0.5, 1:1 and 1:2) was dissolved in deionized water under shaking (100 rpm and 25 °C) for 24 h, followed by centrifugation (12 krcf, 5 min). The resultant supernatant was analyzed by HPLC as above. The FA content was calculated according to the regression equation obtained above.

### 2.5. Bacterial Culture

The selected pathogens *E. coli* (ATCC25922), *P. aeruginosa* (ATCC15692) and *S. aureus* (ATCC29213) were incubated in the Luria–Bertani (LB) culture medium at 37 °C with shaking at 150 rpm. At the exponential growth phase, bacteria suspension was centrifuged (20 °C, 1800 rcf, 15 min) and diluted to a final concentration of ~5 × 10^4^ CFU (colony-forming unit)/mL in fresh LB for further use.

### 2.6. Antibacterial Activity of FA-PC

The antibacterial activity of the FA-PC (molar ratio 1:1) against Gram-negative pathogens *E. coli* and *P. aeruginosa* was examined by CFU counting-based quantification as previously reported [34,35,36]. The FA-PC water solution was prepared the same as above. The phospholipid water solution was also prepared in the same way. For FA water suspension preparation, the FA powder was suspended in water and subjected to the probe sonication (Scientz-IID, Ningbo, China) at 25 W for 5 min (3 s on and 3 s off), followed with continuous shaking (100 rpm and 25 °C) for 24 h.

At the volume ratio of 4:1 (drug solution to bacterial suspension), the FA-PC, FA, phospholipid (equivalent to the content in the FA-PC) and water (control) were mixed with bacterial suspension, respectively. The final concentration of sample equivalent to FA was 20 µg/mL. At predetermined time intervals (6, 12 and 24 h) post-incubation at 37 °C, the bacterial suspension was serially diluted with PBS, and 10 μL of the diluted suspension was inoculated onto LB agar plates. After incubation for 16 h, the CFU grown on the agar plates was counted. 

The concentration-dependent antibacterial activity of the FA-PC against *E. coli* and *P. aeruginosa* was investigated at the concentrations of 5, 10 and 20 µg/mL (equivalent to FA). In addition, the antibacterial activity of the FA-PC against a typical Gram-positive pathogen *S. aureus* was also examined at the concentrations of 1, 2 and 4 µg/mL (equivalent to FA). The antibacterial effect was presented by the loss of viability according to the following equation [1,7]:

Loss of viability (%) = (CFU_Control_ − CFU_Sample_)/CFU_Control_ × 100.

### 2.7. Scanning Electron Microscopy (SEM)

The morphology of *E. coli* and *P. aeruginosa* cells before and after the FA-PC treatment was examined by SEM (SU8200, Hitachi Ltd., Tokyo, Japan). Briefly, the clean round cover glasses were placed in the 24-well culture plates in advance, followed by adding bacterial suspensions and samples. After treatment, the cover glasses were taken out carefully and the bacteria cells were fixed with 2.5% glutaraldehyde at 4 °C for 3 h, followed by gradient dehydration in 30%, 50%, 75% and 100% ethanol solutions (10 min in each). After air-drying at room temperature, the specimens were coated with gold and examined by SEM at a voltage of 10 kV.

### 2.8. Fluorescence Analysis of Intracellular Drug–Phospholipid Complex

In order to further demonstrate the intracellular delivery of the FA-PC, a hydrophobic green fluorescent probe coumarin-6 (C6) was used to replace FA to form the complex with phospholipid (that is C6-PC), and the intracellular C6-PC was examined by confocal laser scanning microscopy (CLSM). Briefly, the C6-PC at the molar ratio of 1:1 was prepared using the same method as the FA-PC. The clean cover glasses were placed in the wells of 24-well culture plates in advance. Afterward, the C6-PC (160 µg/mL), C6 (160 µg/mL, dissolved in 0.5% DMSO) and water (control) were mixed with *E. coli* suspension (1 × 10^6^ CFU/mL) in the plate wells and incubated at 37 °C for 3 h. The cover glasses were carefully taken out and the treated bacteria cells were fixed with 2.5% glutaraldehyde at 4 °C for 3 h, followed by gradient dehydration in 30%, 50%, 75% and 100% ethanol solutions (10 min in each). At last, the bacteria cells were stained with DAPI for 10 min. The resultant specimens were examined by an Olympus FV3000RS confocal microscope (Olympus Corporation, Tokyo, Japan). The fluorescence intensity was analyzed by Image J (Version Java 1.8.0).

### 2.9. Competition Inhibition of FA-PC Delivery by Extra Phospholipid

In order to investigate the transportation mechanisms of the FA-PC, the antibacterial activity of the FA-PC and the intracellular delivery of the C6-PC in the presence of extra phospholipids were investigated. In brief, *E. coli* suspension was treated with the FA-PC (10 µg/mL) in the presence of different amounts of extra phospholipid (0, 1, 2 and 4 mg/mL). At predetermined time intervals (6 and 12 h), the *E. coli* cells were serially diluted with PBS, and 10 μL of the diluted bacterial suspensions were inoculated onto LB agar plates. After further incubation for 16 h, the CFUs grown on agar plates were counted, and the bacteria viability loss was calculated according to the equation above. 

In addition, *E. coli* suspension was also treated with the C6-PC (160 µg/mL) in the presence of different amounts of extra phospholipid (0, 1, 2 and 4 mg/mL). The bacteria treated with water served as a control. After incubation at 37 °C for 3 h, the bacteria cells were subjected to fixing, dehydration and DAPI staining, the same as above. The resultant specimens were examined by CLSM to demonstrate the intracellular delivery of the C6-PC.

### 2.10. Statistics

In this work, all the experiments were performed in triplicate at least, and all the data were presented as mean ± standard deviation (SD). Differences between groups were assessed using a *t*-test or one-way ANOVA (GraphPad Prism 9.0). The difference was considered statistically significant when *p* < 0.05. 

## 3. Results and Discussion

### 3.1. Preparation and Characterization of FA-PC

**Figure 1:**
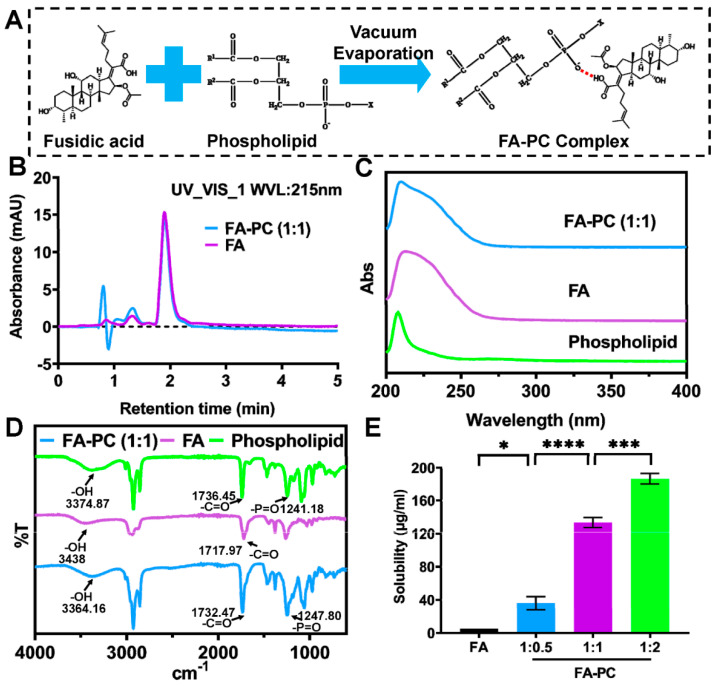
Preparation and characterization of FA-PC. (**A**) Schematic illustration of the preparation of FA-PC. (**B**) HPLC graphs of FA-PC (molar ratio 1:1) and FA. (**C**) UV spectra and (**D**) FTIR spectra of FA-PC (molar ratio 1:1), FA and phospholipid. (**E**) Water-solubility of FA-PC at different molar ratios examined by HPLC. Data are presented as mean ± SD (*n* = 3). Statistical analysis: * *p* < 0.05; *** *p* < 0.001; **** *p* < 0.0001.

The FA-PC was simply synthesized via co-dissolving FA and phospholipid in absolute ethanol, followed by magnetic stirring and rotary evaporation under vacuum (Figure 1A). The HPLC results (Figure 1B) show that the FA-PC (molar ratio 1:1) and FA have the same peak shape and the same retention time (retention time 1.9 min). The FA-PC and FA also have a similar UV absorption spectrum (Figure 1C). FTIR spectra show that the stretching vibration of –OH significantly shifted from 3438 cm^−1^ in FA to 3364 cm^−1^ in the FA-PC (Figure 1D), indicating the formation of a hydrogen bond between the FA and phospholipids [37]. Similar HPLC graphs and UV and FTIR spectra were also presented by the FA-PC at the other molar ratios (1:0.5 and 1:2, Figure S1). These results indicate that the FA-PC is a physical complex of FA with PC but not a new compound [32].

Favorable water solubility of a drug is highly important for its convenient delivery and clinical applications. It is known that the water solubility of FA is only 5.2 μg/mL [24]. Such a low solubility may limit the efficacy and wide application of FA. Many previous works have reported the enhanced solubility of various drugs upon the formation of a phospholipid complex [31,33,38]. Herein, we determined the water solubility of the FA-PC using HPLC-based calibration. The developed HPLC method shows a great separation for FA, and the linear regression equation has a high linear correlation coefficient (R^2^ = 0.9999) (Figure S2). As shown in Figure 1E, the poor water solubility of FA was significantly enhanced to 36.14 μg/mL by the FA-PC (1:0.5), to 133.1 μg/mL by the FA-PC (1:1) and to 186.7 μg/mL by the FA-PC (1:2). Such an enhancement in water solubility may substantially facilitate the antibacterial application of FA.

### 3.2. Antibacterial Activity of FA-PC

**Figure 2:**
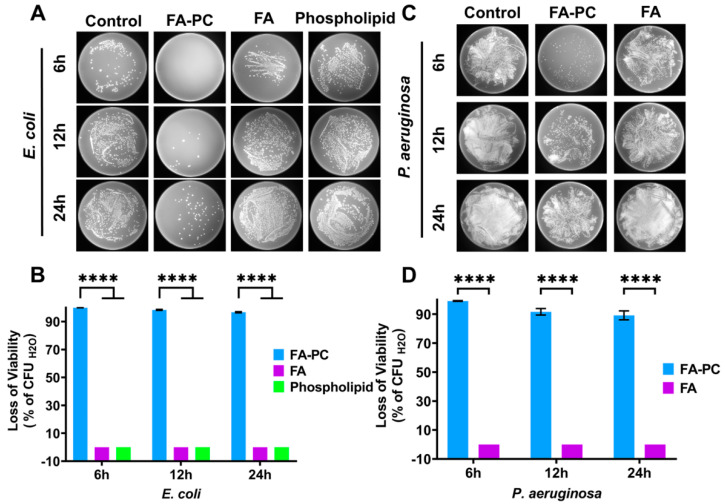
Antibacterial effect of FA-PC (1:1, 20 µg/mL) against *E. coli* and *P. aeruginosa* upon incubation for 6, 12 and 24 h. (**A**) Photos of *E. coli* colonies grown on LB agar plates after treatment with water (control), FA-PC, FA and phospholipid. (**B**) CFU counting-based quantification of viability loss of *E. coli*. (**C**) Photos of *P. aeruginosa* colonies grown on LB agar plates. (**D**) CFU counting-based quantification of viability loss of *P. aeruginosa*. Data are presented as mean ± SD (*n* = 3). Statistical significance: **** *p* < 0.0001.

In order to examine whether the FA-PC is able to reverse the natural resistance of Gram-negative bacteria to FA, the antibacterial activity of the FA-PC (molar ratio 1:1) against *E. coli* and *P. aeruginosa* was investigated. As shown in Figure 2A, the *E. coli* colonies grown on LB agar plates increased rapidly in the control group from 6 to 24 h (i.e., water), indicating that *E. coli* cells had normal growth under the culture conditions. The same rapid growth of *E. coli* was also found in FA (20 µg/mL) and phospholipid groups. It means that free FA or phospholipid alone has no inhibition effect on the growth of *E. coli*. In the presence of the FA-PC (20 µg/mL), however, almost no *E. coli* colonies were found at 6 h and only a few colonies appeared at 12 and 24 h post-incubation. It implies that the growth of *E. coli* was substantially inhibited by the FA-PC. The CFU counting-based quantification further showed that the FA-PC caused an *E. coli* viability loss of 99.9% at 6 h, a 98.4% loss at 12 h and a 96.7% loss at 24 h, while free FA or phospholipid caused no viability loss at all times (Figure 2B). These results indicate that the FA-PC has great antibacterial activity against *E. coli*.

In the case of *P. aeruginosa*, almost the same results were found. *P. aeruginosa* colonies grown on LB agar plates increased rapidly in both control and FA groups, but there were far fewer colonies in the FA-PC group (Figure 2C). It implies that the growth of *P. aeruginosa* is not inhibited by free FA but is highly inhibited by the FA-PC. The CFU counting-based quantification further showed that the FA-PC caused a *P. aeruginosa* viability loss of 99.1% at 6 h, a 91.6% loss at 12 h and an 89.2% loss at 24 h, while free FA caused no viability loss at all (Figure 2D). These results indicate that the FA-PC has great antibacterial activity against *P. aeruginosa*. In addition, the FA-PC also showed a greater antibacterial activity against Gram-positive pathogen *S. aureus* than free FA (Figure S3). 

**Figure 3:**
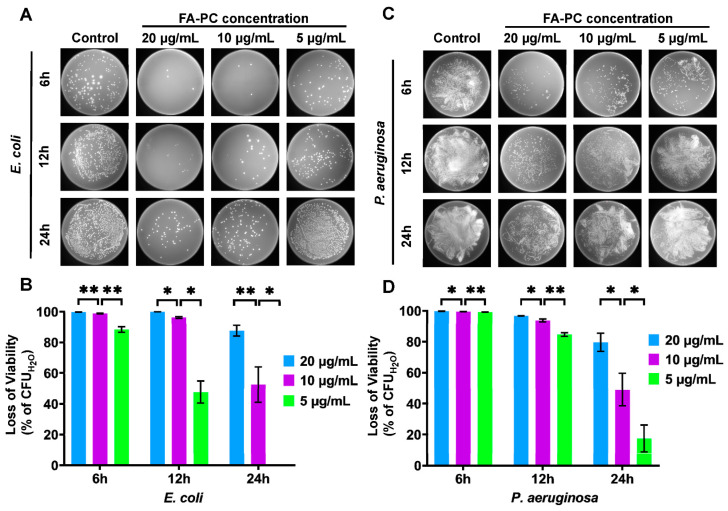
Concentration-dependent antibacterial activity of FA-PC (20, 10 and 5 µg/mL) against *E. coli* and *P. aeruginosa*. (**A**) Typical photos of *E. coli* colonies grown on LB agar plates. (**B**) The loss of viability of *E. coli* quantified by CFU counting. (**C**) Typical photos of *P. aeruginosa* colonies grown on LB agar plates. (**D**) The loss of viability of *P. aeruginosa* quantified by CFU counting. Data are presented as mean ± SD (*n* = 3). Statistical analysis: * *p* < 0.05; ** *p* < 0.01.

The concentration-dependent antibacterial activity of the FA-PC (20, 10 and 5 µg/mL) against *E. coli* and *P. aeruginosa* was also investigated. As shown in Figure 3A, the number of colonies of *E. coli* on agar plates increases with decreasing FA-PC concentrations at each time interval. The CFU counting-based quantification provided more useful information (Figure 3B). At 20 µg/mL, the FA-PC showed very strong anti-*E. coli* activity until 12 h (causing >99% viability loss) and a marginally reduced antibacterial effect at 24 h. At 10 µg/mL, the FA-PC still showed strong anti-*E. coli* activity until 12 h (causing 98.8% and 96.2% viability loss at 6 and 12 h, respectively) and only weak bacteriostatic effect at 24 h (causing 52.4% viability loss). At 5 µg/mL, the FA-PC showed a strong bacteriostatic effect against *E. coli* at 6 h (causing 88.3% viability loss), maintained a small effect at 12 h (causing 47.6% viability loss) but completely lost its efficacy at 24 h. 

In the case of *P. aeruginosa*, similar concentration-dependent antibacterial results were found (Figure 3C,D). The *P. aeruginosa* colonies grown on LB agar plates increased significantly with decreasing concentration of the FA-PC (Figure 3C). More detailed information can be found in the CFU counting-based quantification (Figure 3D). At 20 µg/mL, the FA-PC showed very strong anti-*P. aeruginosa* activity until 12 h (causing 99.7% and 96.7% viability loss at 6 and 12 h, respectively) and a significantly reduced antibacterial effect at 24 h (causing <90% viability loss). At 10 µg/mL, the FA-PC still showed strong anti-*P. aeruginosa* activity until 12 h (causing 99.4% and 93.8% viability loss at 6 and 12 h, respectively) and only a weak bacteriostatic effect at 24 h (causing 59.6% viability loss). At 5 µg/mL, the FA-PC showed a strong antibacterial effect against *P. aeruginosa* at 6 h (causing 99.3% viability loss), a reduced effect at 12 h (causing 84.6% viability loss) and completely lost its efficacy at 24 h (viability loss < 40%). In addition, the FA-PC also showed concentration-dependent antibacterial activity against the Gram-positive pathogen *S. aureus* (Figure S4).

The concentration-dependent and time-dependent antibacterial activity of the FA-PC can provide an important guideline for its practical use in the treatment of Gram-negative bacterial infections. For example, the FA-PC can be administered once a day at the high dosage (i.e., exposure concentration is ~20 µg/mL), twice a day at the medium dosage (i.e., exposure concentration is ~10 µg/mL) and three times a day at the low dosage (i.e., exposure concentration is ~5 µg/mL). 

All these results shown in Figure 2 and Figure 3 clearly show that free FA has no antibacterial activity against *E. coli* or *P. aeruginosa*. However, the FA-PC has great antibacterial activity against those Gram-negative pathogens that have a natural resistance to free FA. This successful reversing of Gram-negative pathogens’ resistance to FA via forming an FA-PC has never been reported before. 

### 3.3. Morphology Changes in Bacteria Cells

**Figure 4:**
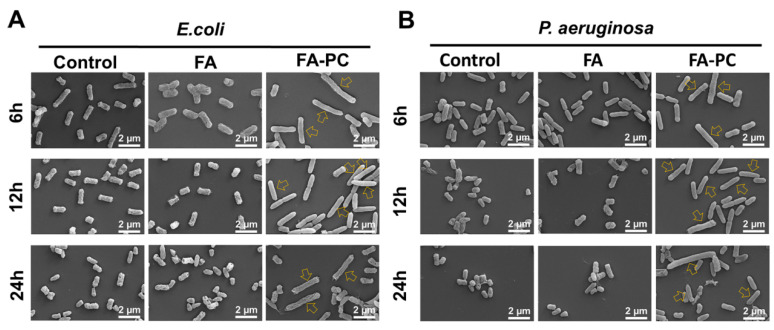
Representative SEM images of (**A**) *E. coli* and (**B**) *P. aeruginosa* morphology after treatment with FA and FA-PC for 6, 12 and 24 h. Hollow arrows indicate the significantly elongated bacteria cells. Scale bar: 2 μm.

The morphology changes in Gram-negative pathogens *E. coli* and *P. aeruginosa* after treatment with the potent FA formulation have never been reported. Herein, we used SEM to examine the morphology changes in these pathogens after treatment with the FA-PC. As shown in Figure 4A, the *E. coli* cells in the control groups show the typical rod shape [39]. After treatment with FA, the cell shape had almost no changes, indicating the ineffectiveness of FA. Interestingly, there are a lot of obviously elongated *E. coli* cells after treatment with the FA-PC (hollow arrows indicate the significantly elongated bacteria cells, Figure 4A). Such an elongation implies that the cell division of *E. coli* is inhibited by the FA-PC, which may be relevant to the antibacterial mechanism of FA (binding to elongation factor G [13,14]). In the case of *P. aeruginosa*, the free FA also had little effect on the cell morphology, but the significant elongation of *P. aeruginosa* cells was found after treatment with the FA-PC (hollow arrows indicate the significantly elongated bacteria cells, Figure 4B), the same as that found in *E. coli* cells. 

These SEM images demonstrate the FA-PC-caused elongation of Gram-negative bacteria cells for the first time and support the antibacterial quantitative data in Figure 2 and Figure 3. As we know, the action target of FA is the elongation factor G located on the ribosome in the cytoplasm [11,12,13,14]. In this regard, we have reason to believe that the FA-PC is successfully delivered into Gram-negative bacteria cells via transmembrane transportation, and this needs further investigation.

### 3.4. Intracellular Delivery of Drug–Phospholipid Complex

**Figure 5:**
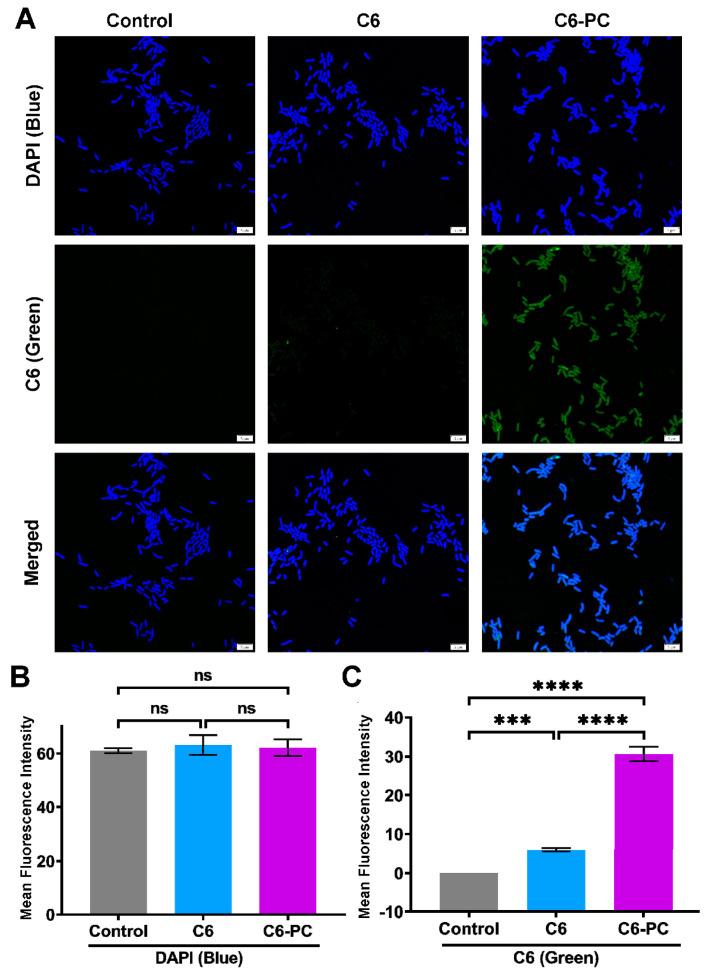
Analysis of intracellular drug delivery into *E. coli* cells using CLSM. (**A**) CLSM images of *E. coli* cells treated with water (control), free C6 and C6-PC. Scale bar: 5 µm. Image J-based fluorescence intensity analysis of (**B**) DAPI (blue) and (**C**) C6 (green). Data are presented as mean ± SD (*n* = 3). Statistical significance: ns, nonsignificant; *** *p* < 0.001; **** *p* < 0.0001.

The results shown above suggest that the FA-PC may be delivered into the cytoplasm of Gram-negative bacteria cells via transmembrane transportation, so as to demonstrate its great antibacterial activity. Herein, we used the hydrophobic C6 as a green fluorescence probe to form the C6-PC and tracked the intracellular C6-PC in *E. coli* cells using CLSM. As shown in Figure 5A, *E. coli* cells in all groups show strong blue fluorescence, indicating the successful staining by DAPI. The cells in the control group show no green fluorescence and those treated with free C6 only show weak green fluorescence. In contrast, strong green fluorescence can be found in the *E. coli* cells treated with the C6-PC. The fluorescence intensity analysis shows that all groups have comparable blue fluorescence intensity (Figure 5B), indicating the comparable number of living *E. coli* cells, which excludes the influence of cell number on the following analysis of green fluorescence intensity. As shown in Figure 5C, the bacteria cells treated with the C6-PC have a green fluorescence intensity of 30.6, significantly higher than the green fluorescence intensity of 6.0 seen following treatment with free C6 (5-fold higher). It implies that only a little free C6 was delivered into *E. coli* cells, but the successful intracellular delivery was realized in the form of the C6-PC. The CLSM images provide a visualized result to further confirm successful drug delivery into Gram-negative bacteria cells in the form of the drug–phospholipid complex.

According to the antibacterial results (Figure 2, Figure 3 and Figure 4) and the intracellular imaging analysis (Figure 5), phospholipids must play essential roles in reversing the natural resistance of Gram-negative pathogens to FA. It is undoubted that the exogenous phospholipid in the FA-PC occupies the reaction site of FA already and thus highly inhibits the interaction of FA with the endogenous membrane phospholipids, allowing the efficient transmembrane transport of FA in the form of the FA-PC via simple diffusion. But it is unknown whether there is a co-existence of phospholipid-associated facilitated diffusion. 

### 3.5. Compromised Antibacterial Activity of FA-PC by Extra Phospholipid

**Figure 6:**
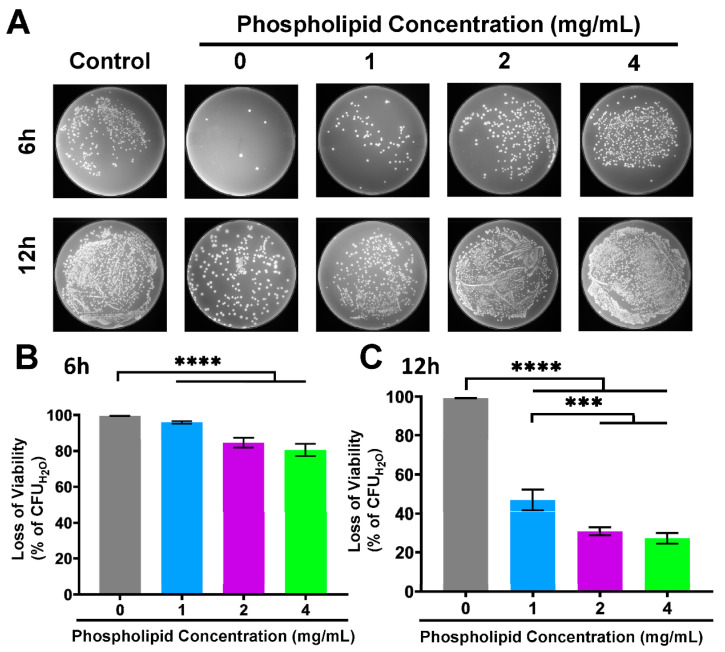
The antibacterial activity of FA-PC against *E. coli* in the presence of extra phospholipid (1, 2 and 4 mg/mL). (**A**) Typical photos of *E. coli* colonies grown on LB agar plates. (**B**) CFU counting-based quantification of viability loss of *E. coli* at 6 h. (**C**) CFU counting-based quantification of viability loss of *E. coli* at 12 h. Data are presented as mean ± SD (*n* = 3). Statistical significance: *** *p* < 0.001; **** *p* < 0.0001.

In order to preliminarily disclose whether the phospholipid-associated facilitated diffusion exists, we investigate the effect of extra phospholipid (1, 2 and 4 mg/mL) on the antibacterial activity of the FA-PC against *E. coli*. As shown in Figure 6A, the number of *E. coli* colonies grown on LB agar plates at 6 h post-incubation increases with increasing phospholipid concentration, and this phenomenon is more obvious at 12 h. The CFU-based quantification shows that the anti-*E. coli* activity of the FA-PC at 6 h post-incubation decreased from 99.5% to 95.8%, 84.5% and 80.5% with the addition of 1, 2 and 4 mg/mL of extra phospholipid, respectively (Figure 6B). At 12 h post-incubation, the presence of extra phospholipid almost caused the FA-PC to completely lose its anti-*E. coli* activity, with antibacterial activity remarkably decreased from 99.5% down to less than 50% (Figure 6C).

It is clear that the extra phospholipid significantly compromised the antibacterial activity of the FA-PC in a concentration-dependent manner. On the one hand, the exogenous phospholipid prevents FA from being captured by membrane phospholipids and reverses the natural resistance of Gram-negative bacteria to FA via forming an FA-PC. On the other hand, the extra exogenous phospholipid inhibits the antibacterial activity of the FA-PC. It suggests that competition may exist between the extra phospholipid and the phospholipid in the FA-PC and that phospholipid-related transmembrane transportation may be involved in the intracellular delivery of the FA-PC.

### 3.6. Inhibited Intracellular Delivery of Drug–Phospholipid Complex by Extra Phospholipid

**Figure 7:**
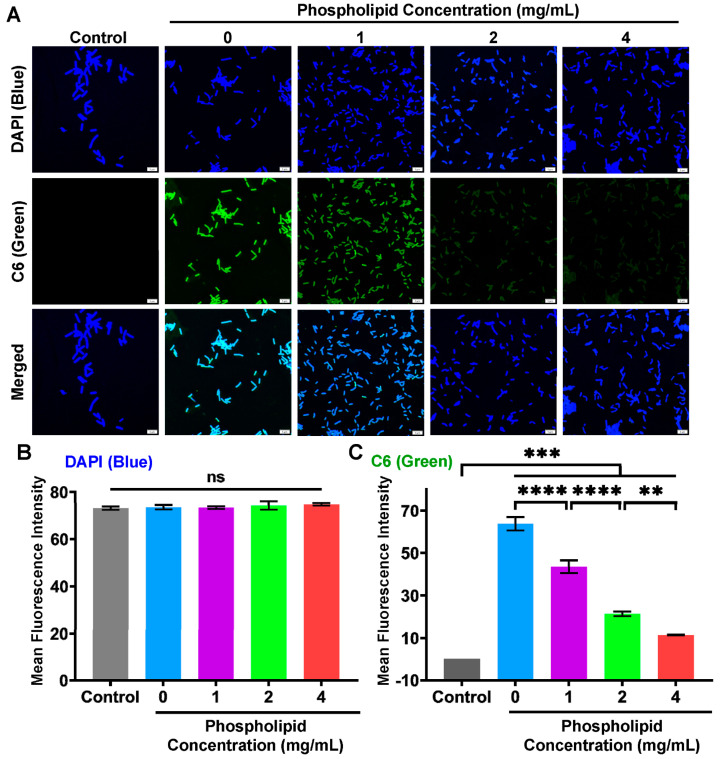
Analysis of intracellular drug delivery into *E. coli* cells in the presence of extra phospholipid (1, 2 and 4 mg/mL) using CLSM. (**A**) CLSM images of *E. coli* cells treated with water (control) and C6-PC in the absence and presence of extra phospholipid. Scale bar: 5 µm. Image J-based fluorescence intensity analysis of (**B**) DAPI (blue) and (**C**) C6 (green). Data are presented as mean ± SD (*n* = 3). Statistical significance: ns, nonsignificant; ** *p* < 0.01; *** *p* < 0.001; **** *p* < 0.0001.

In order to further examine the effect of extra phospholipid on the intracellular delivery of the FA-PC, we investigate the intracellular imaging of the C6-PC in the presence of extra phospholipid (1, 2 and 4 mg/mL) using CLSM. As shown in Figure 7A, *E. coli* cells in all groups show comparable strong blue fluorescence (DAPI). As expected, the green fluorescence, which indicates the intracellular amount of the C6-PC, is strongest in the C6-PC without extra phospholipid and becomes darker with increasing phospholipid concentration. The Image J-based fluorescence intensity analysis shows that the bacteria cells in all groups have comparable DAPI staining (Figure 7B). In contrast, the green fluorescence intensity is affected by the concentration of extra phospholipid. In detail, the green fluorescence intensity significantly decreased from 63.7 at 0 mg/mL of extra phospholipid to 43.5, 21.3 and 11.4 at 1, 2 and 4 mg/mL of extra phospholipid (Figure 7C). The concentration-dependent green fluorescence intensity further confirms that the intracellular delivery of the FA-PC is significantly inhibited by the extra phospholipid.

The CLSM images in Figure 7 match well with the antibacterial quantitative results shown in Figure 6 and further confirm the existence of phospholipid-associated transmembrane transportation in the intracellular delivery of the FA-PC. But the real mechanisms underlying the intracellular delivery of the FA-PC into Gram-negative pathogens are unclear. A recent report suggests that the MCE (mammalian cell entry) transporter is a key factor facilitating the transmembrane uptake of lipids by *M. smegmatis*, a Gram-negative bacterium relative of *M. tuberculosis* [40]. This may be involved in the intracellular transportation of the FA-PC, but further investigations are required to confirm it.

## 4. Conclusions

In this work, we synthesized a complex of fusidic acid (FA) with phospholipid, namely FA-PC, via simple co-dissolving and solvent evaporation methods. The FA-PC is not a new compound in nature, but a physical composite based on the strong interactions between FA and phospholipid. The FA-PC showed a strong antibacterial activity against two representative Gram-negative pathogens that have intrinsic resistance to free FA. In detail, the FA-PC (molar ration 1:1, equivalent to 20 µg/mL FA) caused 99.9% viability loss of *E. coli* and 99.1% viability loss of *P. aeruginosa* after incubation for 6 h. In the meantime, the FA-PC maintained great antibacterial activity against the Gram-positive pathogen. The SEM (scanning electron microscopy) images demonstrated that the morphology of *E. coli* and *P. aeruginosa* cells was significantly elongated after treatment with the FA-PC. Furthermore, a fluorescent probe C6 was complexed with phospholipid, and CLSM (confocal laser scanning microscopy) clearly demonstrated that the C6-PC was successfully delivered into *E. coli* cells. 

Overall, our findings demonstrate the great intracellular delivery and antibacterial activity of FA against Gram-negative pathogens in the form of the FA-PC. This is the first report regarding the successful reversing of the natural drug resistance of Gram-negative pathogens to FA via forming an FA-PC. On the one hand, the exogenous phospholipid in the FA-PC prevents FA from capture by bacterial membrane phospholipids and facilitates the intracellular delivery of FA. On the other hand, the extra exogenous phospholipid also inhibits the intracellular delivery of the FA-PC, suggesting that phospholipid-related transmembrane transportation may be involved in the intracellular delivery of the FA-PC. But the real mechanisms are unknown and need further investigation. In conclusion, our findings provide a platform to reverse transmembrane delivery-related drug resistance. The ready availability of phospholipids and the simple preparation allows it to have great potential for clinical use. 

## Data Availability

Data are contained within the article and supplementary materials.

## Supplementary Materials

The following supporting information can be downloaded at: https://www.mdpi.com/article/10.3390/bioengineering11020177/s1, Figure S1. HPLC graphs of (**A**) FA-PC (1: 0.5) and (**B**) FA-PC (1: 2). The FTIR spectra of (**C**) FA-PC (1: 0.5) and (**D**) FA-PC (1: 2). UV spectra of (**E**) FA-PC (1: 0.5) and (**F**) FA-PC (1: 2); Figure S2. The HPLC graphs of FA standards (**A**) and the HPLC-based calibration and linear regression (**B**). Figure S3. Antibcaterial ability of FA-PC and FA (4 µg/mL) against *S. aureus*. (**A**) Typical photos of *S. aureus* on agar plates after treatment for 6 h. (**B**) The loss of viability of *S. aureus*. Data are presented as mean ± SD (*n* = 3). Statistical significance: **** *p* < 0.0001. Figure S4. Concentration-dependent antibacterial ability of FA-PC (4, 2 and 1 µg/mL) against *S. aureus*. (**A**) Typical photos of *S. aureus* grown on agar plates. (**B**) The loss of viability of *S. aureus*. Data are presented as mean ± SD (*n* = 3). Statistical significance: ns, non-significant; ** *p* < 0.01; **** *p* < 0.0001.

## References

[1] Gao Y., Dong Y., Yang S., Mo A., Zeng X., Chen Q., Peng Q. (2022). Size-dependent photothermal antibacterial activity of Ti_3_C_2_T*_x_* MXene nanosheets against methicillin-resistant *Staphylococcus aureus*. J. Colloid Interface Sci..

[2] Tan Y., Su J., Fu M., Zhang H., Zeng H. (2023). Recent Advances in Phage-Based Therapeutics for Multi-Drug Resistant *Acinetobacter baumannii*. Bioengineering.

[3] Huang W., Meng L., Chen Y., Dong Z., Peng Q. (2022). Bacterial outer membrane vesicles as potential biological nanomaterials for antibacterial therapy. Acta Biomater..

[4] Zhang Y., Wang D., Liu F., Sheng S., Zhang H., Li W., Li Y., Tian H. (2022). Enhancing the drug sensitivity of antibiotics on drug-resistant bacteria via the photothermal effect of FeTGNPs. J. Control Release.

[5] Wang T., Li Y., Liu Y., Xu Z., Wen M., Zhang L., Xue Y., Shang L. (2023). Highly biocompatible Ag nanocluster-reinforced wound dressing with long-term and synergistic bactericidal activity. J. Colloid Interface Sci..

[6] Wang X., Sun X., Liu W., Li H., Wang J., Wang D. (2024). Amino acid-mediated amorphous copper sulphide with enhanced photothermal conversion efficiency for antibacterial application. J. Colloid Interface Sci..

[7] Yu C., Sui S., Yu X., Huang W., Wu Y., Zeng X., Chen Q., Wang J., Peng Q. (2022). Ti3C2Tx MXene loaded with indocyanine green for synergistic photothermal and photodynamic therapy for drug-resistant bacterium. Colloids Surf. B Biointerfaces.

[8] Ni J., Guo M., Cao Y., Lei L., Liu K., Wang B., Lu F., Zhai R., Gao X., Yan C. (2019). Discovery, synthesis of novel fusidic acid derivatives possessed amino-terminal groups at the 3-hydroxyl position with anticancer activity. Eur. J. Med. Chem..

[9] Long J., Ying T., Zhang L., Yu T., Wu J., Liu Y., Li X., You G., Zhang L., Bi Y. (2022). Discovery of fusidic acid derivatives as novel STING inhibitors for treatment of sepsis. Eur. J. Med. Chem..

[10] Marian E., Tita B., Duteanu N., Vicas L., Ciocan S., Jurca T., Antal L., Tica O., Mureşan M., Pallag A. (2020). Antimicrobial activity of fusidic acid inclusion complexes. Int. J. Infect. Dis..

[11] Chavez M.G., Garcia A., Lee H.Y., Lau G.W., Parker E.N., Komnick K.E., Hergenrother P.J. (2021). Synthesis of Fusidic Acid Derivatives Yields a Potent Antibiotic with an Improved Resistance Profile. ACS Infect. Dis..

[12] Long J., Ji W., Zhang D., Zhu Y., Bi Y. (2021). Bioactivities and Structure–Activity Relationships of Fusidic Acid Derivatives: A Review. Front. Pharm..

[13] Romaru J., Limelette A., Lebrun D., Bonnet M., Garnier V.V., N’Guyen Y. (2022). Fusidic acid in a tertiary hospital: An observational study focusing on prescriptions, tolerance and susceptibility of *Staphylococcus* and *Cutibacterium* spp. strains from bone samples. Eur. J. Clin. Microbiol. Infect. Dis..

[14] Singh V., Dziwornu G.A., Mabhula A., Chibale K. (2021). Rv0684/fusA1, an Essential Gene, Is the Target of Fusidic Acid and Its Derivatives in *Mycobacterium tuberculosis*. ACS Infect. Dis..

[15] Borg A., Pavlov M., Ehrenberg M. (2016). Mechanism of fusidic acid inhibition of RRF- and EF-G-dependent splitting of the bacterial post-termination ribosome. Nucleic Acids Res..

[16] Ayyub S.A., Lahry K., Dobriyal D., Mondal S., Varshney U. (2018). Antimicrobial activity of fusidic acid in *Escherichia coli* is dependent on the relative levels of ribosome recycling factor and elongation factor G. FEMS Microbiol. Lett..

[17] Borg A., Holm M., Shiroyama I., Hauryliuk V., Pavlov M., Sanyal S., Ehrenberg M. (2015). Fusidic acid targets elongation factor G in several stages of translocation on the bacterial ribosome. J. Biol. Chem..

[18] Belardinelli R., Rodnina M.V. (2017). Effect of Fusidic Acid on the Kinetics of Molecular Motions During EF-G-Induced Translocation on the Ribosome. Sci. Rep..

[19] Biedenbach D.J., Rhomberg P.R., Mendes R.E., Jones R.N. (2010). Spectrum of activity, mutation rates, synergistic interactions, and the effects of pH and serum proteins for fusidic acid (CEM-102). Diagn. Microbiol. Infect. Dis..

[20] Nicolosi D., Cupri S., Genovese C., Tempera G., Mattina R., Pignatello R. (2015). Nanotechnology approaches for antibacterial drug delivery: Preparation and microbiological evaluation of fusogenic liposomes carrying fusidic acid. Int. J. Antimicrob. Agents.

[21] Chen S., Zhou P., Wu C., Wang J., Zhou Y., Zhang J., Wang B., Zhao H., Rao L., Li M. (2023). Polymyxin B and fusidic acid, a novel potent synergistic combination against *Klebsiella pneumoniae* and *Escherichia coli* isolates with polymyxin B resistance. Front. Microbiol..

[22] Helle A., Makitalo J., Huhtanen J., Holopainen J.M., Wiedmer S.K. (2008). Antibiotic fusidic acid has strong interactions with negatively charged lipid membranes: An electrokinetic capillary chromatographic study. Biochim. Biophys. Acta.

[23] Falck E., Hautala J.T., Karttunen M., Kinnunen P.K., Patra M., Saaren-Seppala H., Vattulainen I., Wiedmer S.K., Holopainen J.M. (2006). Interaction of fusidic acid with lipid membranes: Implications to the mechanism of antibiotic activity. Biophys. J..

[24] Gilchrist S.E., Rickard D.L., Letchford K., Needham D., Burt H.M. (2012). Phase separation behavior of fusidic acid and rifampicin in PLGA microspheres. Mol. Pharm..

[25] Ma J., Chen D., Li Y., Chen Y., Liu Q., Zhou X., Qian K., Li Z., Ruan H., Hou Z. (2018). Zinc phthalocyanine-soybean phospholipid complex based drug carrier for switchable photoacoustic/fluorescence image, multiphase photothermal/photodynamic treatment and synergetic therapy. J. Control Release.

[26] Ebada H.M.K., Nasra M.M.A., Elnaggar Y.S.R., Abdallah O.Y. (2021). Novel rhein-phospholipid complex targeting skin diseases: Development, in vitro, ex vivo, and in vivo studies. Drug Deliv. Transl. Res..

[27] Zhao X., Shi C., Zhou X., Lin T., Gong Y., Yin M., Fan L., Wang W., Fang J. (2019). Preparation of a nanoscale dihydromyricetin-phospholipid complex to improve the bioavailability: In vitro and in vivo evaluations. Eur. J. Pharm. Sci..

[28] Peng Q., Gong T., Zuo J., Liu J., Zhao D., Zhang Z. (2008). Enhanced the oral bioavailability of salvianolic acid B by phospholipid complex loaded nanoparticles. Pharmazie.

[29] Peng Q., Zhang Z.-R., Sun X., Zuo J., Zhao D., Gong T. (2010). Mechanisms of Phospholipid Complex Loaded Nanoparticles Enhancing the Oral Bioavailability. Mol. Pharm..

[30] Peng Q., Yang Y.J., Zhang T., Wu C.Y., Yang Q., Sun X., Gong T., Zhang L., Zhang Z.R. (2013). The implantable and biodegradable PHBHHx 3D scaffolds loaded with protein-phospholipid complex for sustained delivery of proteins. Pharm. Res..

[31] Peng Q., Zhang Z.R., Gong T., Chen G.Q., Sun X. (2012). A rapid-acting, long-acting insulin formulation based on a phospholipid complex loaded PHBHHx nanoparticles. Biomaterials.

[32] Li Y., Yin H., Wu C., He J., Wang C., Ren B., Wang H., Geng D., Zhang Y., Zhao L. (2023). Preparation and in vivo evaluation of an intravenous emulsion loaded with an aprepitant-phospholipid complex. Drug Deliv..

[33] Telange D.R., Sohail N.K., Hemke A.T., Kharkar P.S., Pethe A.M. (2021). Phospholipid complex-loaded self-assembled phytosomal soft nanoparticles: Evidence of enhanced solubility, dissolution rate, ex vivo permeability, oral bioavailability, and antioxidant potential of mangiferin. Drug Deliv. Transl. Res..

[34] Dong Y., Liu J., Chen Y., Zhu T., Li Y., Zhang C., Zeng X., Chen Q., Peng Q. (2023). Photothermal and natural activity-based synergistic antibacterial effects of Ti3C2Tx MXene-loaded chitosan hydrogel against methicillin-resistant *Staphylococcus aureus*. Int. J. Biol. Macromol..

[35] Wang Y., Xu J., Yu C., Zhou X., Chang L., Liu J., Peng Q. (2023). Prevention of bacterial biofilm formation on orthodontic brackets by non-crosslinked chitosan coating. Int. J. Biol. Macromol..

[36] Wang Y., Gao H., Chang L., Xu J., Zhou X., Zhang C., Peng Q. (2023). Efficient Removal of Dental Plaque Biofilm from Training Typodont Teeth via Water Flosser. Bioengineering.

[37] Chi C., Zhang C., Liu Y., Nie H., Zhou J., Ding Y. (2020). Phytosome-nanosuspensions for silybin-phospholipid complex with increased bioavailability and hepatoprotection efficacy. Eur. J. Pharm. Sci..

[38] Chakravarti R.K., Kaur S., Samal S.K., Kashyap M.C., Sangamwar A.T. (2021). Combination of Phospholipid Complex and Matrix Dispersion. AAPS PharmSciTech.

[39] Lin Y., Xu J., Dong Y., Wang Y., Yu C., Li Y., Zhang C., Chen Q., Chen S., Peng Q. (2023). Drug-free and non-crosslinked chitosan/hyaluronic acid hybrid hydrogel for synergistic healing of infected diabetic wounds. Carbohydr. Polym..

[40] Chen J., Fruhauf A., Fan C., Ponce J., Ueberheide B., Bhabha G., Ekiert D.C. (2023). Structure of an endogenous mycobacterial MCE lipid transporter. Nature.

