# Supplementary material for: Reversing the Natural Drug Resistance of Gram-Negative Bacteria to Fusidic Acid via Forming Drug–Phospholipid Complex
Source: Bioengineering (Basel). 2024 Feb 11;11(2):177. doi: 10.3390/bioengineering11020177 (PMC10885999; doi:10.3390/bioengineering11020177)
Supplement: Supplementary file 1 [file bioengineering-11-00177-s001.zip › bioengineering-2846330-supplementary.pdf]

bioengineering

Supporting Information

Reversing the Natural Drug Resistance of Gram-Negative Bacteria to Fusidic Acid via Forming Drug–Phospholipid Complex

Jianhong Liu †, Xuyang Lai †, Yuanhong Li, Zhuohang Yu, Xuan Wang, Chaoliang Zhang and Qiang Peng *

State Key Laboratory of Oral Diseases & National Center for Stomatology & National Clinical Research Center for Oral Diseases, West China Hospital of Stomatology, Sichuan University, Chengdu 610041, China
* Correspondence: qiangpengzz@scu.edu.cn or lijm2002@163.com
† These authors contributed equally to this work.

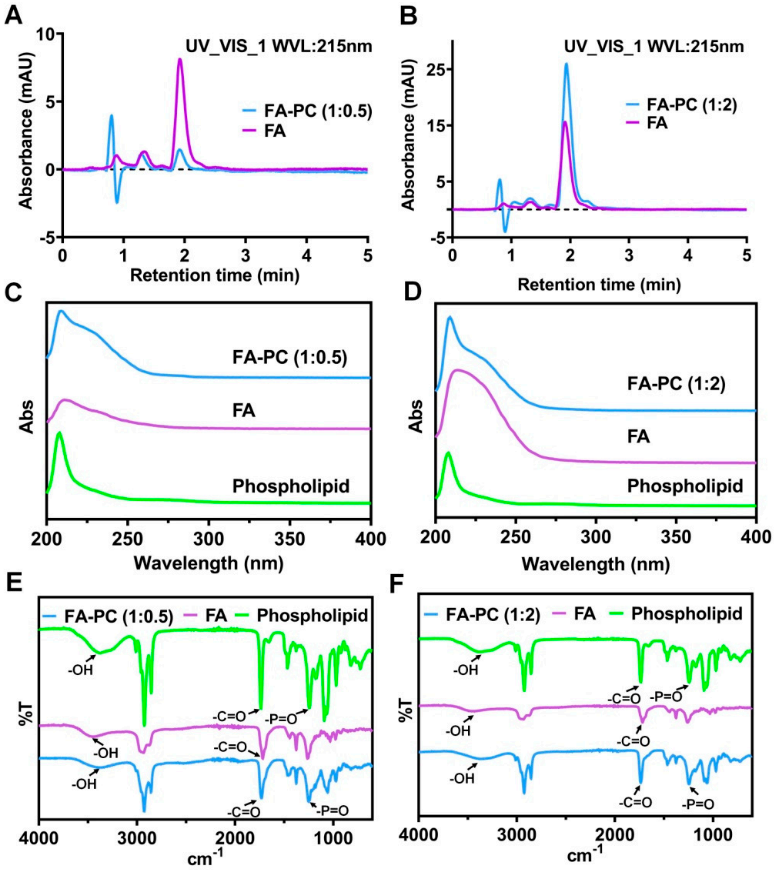

Figure S1. HPLC graphs of (A) FA-PC (1: 0.5) and (B) FA-PC (1: 2). The FTIR spectra of (C) FA-PC (1: 0.5) and (D) FA-PC (1: 2). UV spectra of (E) FA-PC (1: 0.5) and (F) FA-PC (1: 2).

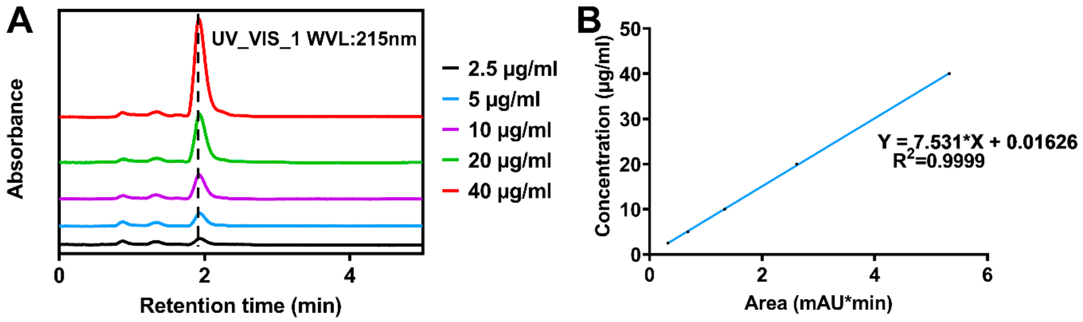

Figure S2. The HPLC graphs of FA standards (A) and the HPLC-based calibration and linear regression (B).

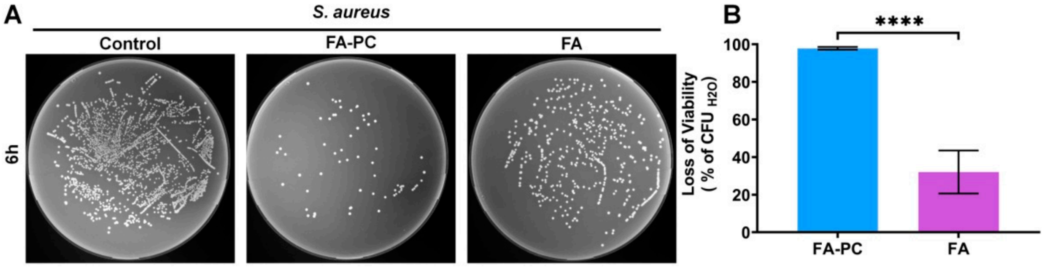

Figure S3. Antibcaterial ability of FA-PC and FA (4 μg/mL) against *S. aureus*. (A) Typical photos of *S. aureus* on agar plates after treatment for 6 h. (B) The loss of viability of *S. aureus*. Data are presented as mean ± SD (n=3). Statistical significance: ****P < 0.0001.

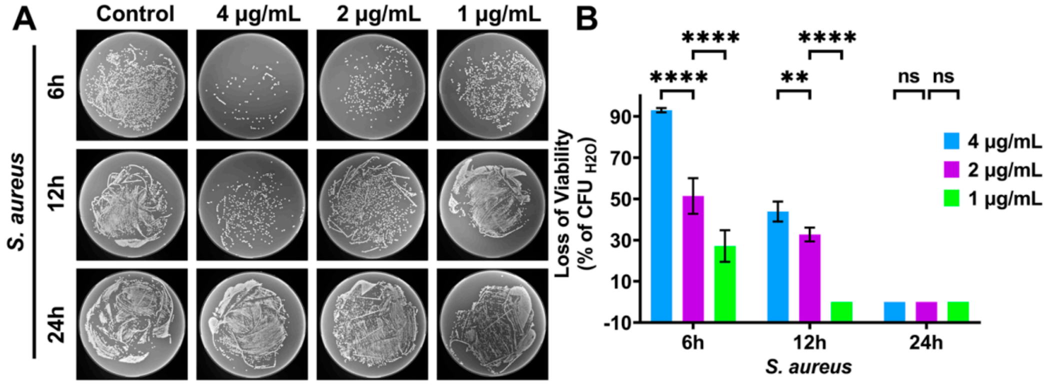

Figure S4. Concentration-dependent antibacterial ability of FA-PC (4, 2 and 1 μg/mL) against *S. aureus*. (A) Typical photos of *S. aureus* grown on agar plates. (B) The loss of viability of *S. aureus*. Data are presented as mean ± SD (n=3). Statistical significance: ns, non-significant; **P < 0.01; ****P < 0.0001.
